# Novel Coating to Minimize Corrosion of Glass-Ceramics for Dental Applications

**DOI:** 10.3390/ma13051215

**Published:** 2020-03-08

**Authors:** Shu-Min Hsu, Fan Ren, Zhiting Chen, Mijin Kim, Chaker Fares, Arthur E. Clark, Dan Neal, Josephine F. Esquivel-Upshaw

**Affiliations:** 1 Department of Restorative Dental Sciences, University of Florida College of Dentistry, Gainesville, FL 32610, USA; mijinkim@ufl.edu (M.K.); BCLARK@dental.ufl.edu (A.E.C.); JESQUIVEL@dental.ufl.edu (J.F.E.-U.); 2 Department of Chemical Engineering, University of Florida, Gainesville, FL 32611, USA; fren@che.ufl.edu (F.R.); zt.chen@ufl.edu (Z.C.); c.fares@ufl.edu (C.F.); 3 Department of Neurosurgery, University of Florida College of Medicine, Gainesville, FL 32610, USA; dneal@ufl.edu

**Keywords:** glass-ceramic, coating, corrosion, weight loss, ion release

## Abstract

The effect of a novel silicon carbide (SiC) coating on the chemical durability of a fluorapatite glass-ceramic veneer was investigated by examining weight loss and ion release levels. The hypothesis that this novel coating will exhibit significant corrosion resistance was tested. Inductively coupled plasma atomic emission spectrometer (ICP) was used for ion concentration determination and scanning electron microscopy (SEM) for surface morphology analyses. Samples were immersed in pH 10 and pH 2 buffer solutions to represent extreme conditions in the oral cavity. Analyses were done at 15 and 30 days. The SiC coated group demonstrated significant reduction in weight loss across all solutions and time points (*p* < 0.0001). Ion release analyses demonstrated either a marginally lower or a significantly lower release of ions for the SiC-coated disks. SEM analysis reveals planarization of surfaces by the SiC-coated group. The surfaces of coated samples were not as corroded as the non-coated samples, which is indicative of the protective nature of these coatings. In conclusion, SiC is a novel coating that holds promise for improving the performance of ceramic materials used for dental applications.

## 1. Introduction

Glass-ceramics undergo corrosion [1,2,3,4,5,6,7,8]. Clinical studies have confirmed this [9] and in vitro studies have shown that different pH levels affect the surface of ceramic adversely. pH 10 was demonstrated to be the most corrosive, followed by pH 7 and pH 2 [1]. The mechanism behind the corrosion process is responsible for the differences in severity. A total dissolution of the network former bonds (Si–Si bonds) occurs in the presence of a pH 10 environment, while an ionic exchange reaction occurs in a pH 2 or acidic environment [1]. pH 7 has an equal amount of ionic exchange and total dissolution that occurs. A recent study [10] concluded that in vitro tests for chemical stability of ceramic products could be underestimating the surface degradation of ceramics by performing tests in constant pH environments only. The oral environment has a dynamic pH that can vacillate from acidic to basic with the constant introduction of food items differing in pH levels and the buffering capacity of saliva. For instance, enamel demineralization and ceramic corrosion can be initiated with all kinds of acidic drinks (e.g., Coca-Cola pH 2.45, Red Bull pH 3.17, orange juice pH 3.74, wines pH 3.34–3.68) [11], food (beef pH 4.1–7.0, lamb pH 5.4–6.7) and fruits (grapefruits pH 3.0–3.3, oranges pH 3.0) [12], or basic substances pH 8–14 (e.g., spinach, soybeans, and antacids) [1]. Eventually, this constant change in pH can decrease the fracture strength of glass-ceramics [13,14] and increase surface roughness [15,16]. This roughening of the restoration can lead to plaque accumulation and increased wear of the opposing enamel [17,18]. Dental restorations should be able to withstand these fluctuations in pH.

Chemical durability of dental materials has been extensively studied because of the importance of this property on the longevity of the restoration. Intrinsic or extrinsic modifications can be used to improve chemical durability. Several studies have demonstrated chemical stability for glass-ceramics can be enhanced by (1) different ratio of compositions; (2) addition of oxides, such as CaO, K_2_O, and Al_2_O_3_; and (3) fluorine and calcium phosphates as part of intrinsic modifications [19,20,21,22,23]. Extrinsic modification can be achieved by producing additional layers on the surface to enhance chemical durability and other physical properties [24,25,26]. Esquivel-Upshaw et al. showed that glazed material has better chemical resistance than non-glazed material [1]. Topateş et al. discussed the effect of different glaze compositions on chemical durability [25]. Rau et al. reported that the chemical resistance and corrosion rate of magnesium alloy were improved with the application of a glass-ceramic coating [26].

Chemical durability is also important because leached ions during corrosion may not be safe. The literature demonstrated the cytotoxicity of materials in ceramic [27,28,29,30]. Elshahawy et al. reported that Zn had the highest cytotoxicity (60% cell viability) to fibroblast cells among the ions released from gold alloys and ceramic crowns in patients’ saliva, followed by Cu > Ag > Pd > Al > Au > Si. The cytotoxicity was not related to the amount of the ion released in this study; Si had the highest released amount, but the least cytotoxicity (90% cell viability). This indicates Si has higher biocompatibility [28]. Another study analyzed the medium where all-ceramic and provisional materials were immersed and found the materials leached to be slightly cytotoxic [30].

Research has shown that silicon carbide (SiC) is a promising ceramic material for biomedical applications [31,32,33]. This material has superior properties including lightweight, high strength, corrosion resistance, and high temperature resistance [31,32,33,34,35,36,37,38,39,40,41]. SiC has been incorporated with ceramic as ceramic composites [34,35,42,43], where increased strength was demonstrated in ceramic crowns infused with SiC fibers [42]. Lithium aluminosilicate glass-ceramic reinforced with SiC fibers and composites of zirconia and SiC particles both exhibited high strength and toughness [34,35].

In addition, SiC demonstrated good biocompatibility. Naji and Harmand reported the cytocompatibility of SiC and amorphous alumina coatings showed that both materials are cytocompatible for human fibroblasts and osteroblasts [44]. Bonaventura et al. explored the biocompatibility of Si and SiC coating to neural stem cells. Their findings demonstrated SiC had higher biocompatibility than Si [45]. Botsoa et al. showed there was no toxicity after the uptake of SiC nanoparticles by 3T3-L1 fibroblasts cells for one week [46]. However, the reported results are controversial because the cytotoxicity was also dose-dependent, morphology-dependent, and structure and surface property-dependent [47,48].

The latest research demonstrated that SiC is a promising coating as dental material, which displayed adjustable color to match the dental shade guide used in clinic and wear resistance [49]. However, the oral environment could be a hostile to dental ceramic materials. For this study, we aim to demonstrate the corrosion resistance of SiC when used as a coating for glass-ceramic veneers. The objective of this study was to test the hypotheses that SiC-coated fluorapatite glass-ceramic veneers will exhibit significant corrosion resistance under different pH environments as a function of weight loss and ion release.

## 2. Materials and Methods

### 2.1. Specimens Preparation

Fluorapatite glass-ceramic disks (Ivoclar Vivadent AG, Schaan, Liechtenstein, 12.6 × 1.3 ± 0.2 mm) were polished through 340 grits, 400 grits. and 600 grits of silicon carbide abrasive paper (Carbimet, Buehler, Lake Bluff, IL, USA) on both sides, cleaned with ethanol under ultrasonic, and rinsed thoroughly using deionized water. The composition of the fluorapatite disks is listed in Table 1 from Ivoclar Vivadent (Schaan, Liechtenstein) scientific documentation.

### 2.2. Coating Fabrication 

Silicon dioxide (SiO_2_) and silicon carbide (SiC) dielectric films were applied to ceramic disks. A total thickness of 250 nm with 20 nm for SiO_2_ and 230 nm for SiC was observed. The SiO_2_/SiC dielectric films were deposited using plasma-enhanced chemical vapor deposition (PECVD, PlasmaTherm 790, Saint Petersburg, FL, USA). Prior to deposition of the dielectric film, a series of cleaning procedures were applied to the glass-ceramic disks to remove debris on the surface produced during polishing. Particle remnants can cause locally induced stresses, which can compromise the adhesion of the coating. Cleaning was achieved through ultra-sonication for 1 min in a solution with a 0.2 ratio between hydrochloric acid and de-ionized water. The solution was changed to just de-ionized water and sonicated for 2 min. The cleaning procedure was repeated three times.

The configuration of PECVD was a parallel plate with a showerhead and a load lock. The substrate holder temperature was maintained at 300 °C, where the SiO_2_/SiC films were deposited on the glass-ceramic disks. Further, 2% silane balanced (SiH_4_) in argon and nitrous oxide (N_2_O) were the precursors for SiO_2_ film. The silane and methane were the precursors for SiC film. The deposition conditions were well calibrated. The deposition rate was 330 Å /min for SiO_2_ and 170 Å/min for SiC. The SiO_2_ was deposited on a glass-ceramic disk and then the SiC was deposited next on SiO_2_/glass-ceramic.

### 2.3. Experimental Design

The two groups in this study are (i) non-coated fluorapatite glass-ceramic disks as reference, and (ii) SiO_2_/SiC coated fluorapatite glass-ceramic disks (SiC-disks). All the disks were dried in an oven (Isotemp Vacuum Oven Model 285A, Fisher Scientific) at a temperature of 100–105 °C for 24 h and cooled in a vacuum desiccator prior to weighing. The weight measurement was performed before and after the corrosion experiment with an accuracy of 0.1 mg (AS60/220.R2 Analytical balance, RADWAG). The disks were constantly immersed in (i) 15 mL of pH 10 (potassium carbonate-potassium borate-potassium hydroxide buffer, SB116-500, Fisher Chemical, Pittsburgh, PA, USA) and (ii) 15 mL of pH 2 buffer solutions (glycine buffer solution, Santa Cruz Biotechnology, Inc., Dallas, TX, USA) in polyethylene centrifuge tubes (Thermo Scientific Nalgene Oak Ridge High-Speed Centriguge Tubes, Thermo Fisher Scientific, Waltham, MA, USA) for 15 and 30 days. Three disks were used for each condition. The tubes were placed in a rotating shaking water bath (water bath shaking TSBS40, Techne, Vernon Hills, IL, USA) at 50 oscillations per minute at 80 °C. After corrosion, the level of ions released into the solution was obtained by inductively coupled plasma atomic emission spectrometer (ICP, 3200RL, PerkinElmer, Waltham, MA, USA). Ions examined were Si, Ca, Zn, and Al.

Statistical analysis was performed using Mann–Whitney U test to determine significant differences in weight loss and ion release between the groups.

### 2.4. Characterizations

The surface morphology of reference and SiC-disks was examined by scanning electron microscopy (SEM). The disks were sputter coated with platinum and then analyzed using field-emission SEM (Nova Nano 430, FEI, Hillsboro, OR, USA). The images were obtained at 5 kV.

The surface composition of reference disks was investigated by X-ray photoelectron spectroscopy instrument (ULVAC-PHI XPS, ULVAC-PHI, Kanagawa, Japan) with Al monochromatised Kα radiation from a 50 W X-ray source.

## 3. Results 

### 3.1. Weight Loss

**Figure 1:**
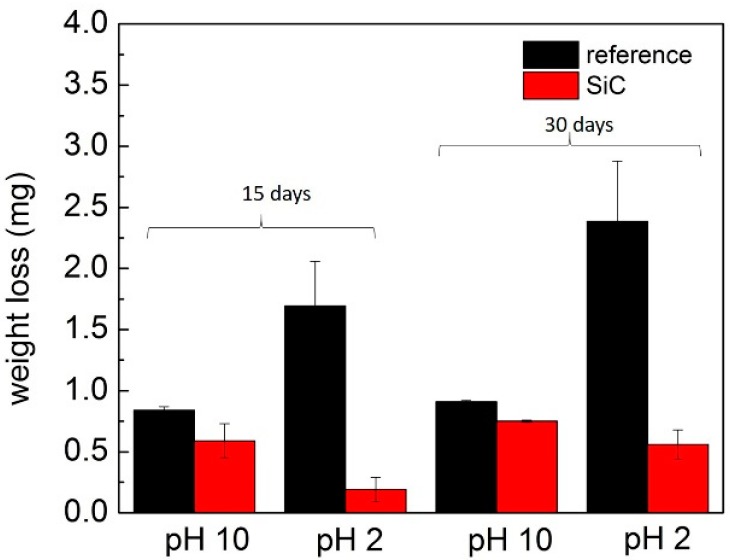
The weight loss of non-coated (ref) and silicon carbide (SiC)-coated disks constantly immersed in pH 10 and pH 2 for 15 and 30 days.

Comparison of weight loss between coated and non-coated dental glass-ceramic disks for different pH solutions and time periods is shown in Figure 1. Weight loss was significantly more for the non-coated groups across all solutions and time points (*p* < 0.0001).

Comparison of weight loss in solutions between coated and non-coated dental glass-ceramic demonstrates there was significantly less weight loss among SiC coated disks both in pH 10 (*p* = 0.005) and pH 2 (*p* = 0.010) compared with their controls.

The SiC coating displayed a protective effect for pH 2 and pH 10 between non-coated and SiC-disks. The weight loss in pH 2 was nine times less at 15 days (*p* = 0.004) and four times less for 30 days (*p* = 0.008) for the coated disks compared with the non-coated disks. The weight loss of coated disks was slightly less in pH 10 than the non-coated disks after 30 days’ immersion (*p* = 0.076).

### 3.2. ICP Analysis

**Figure 2:**
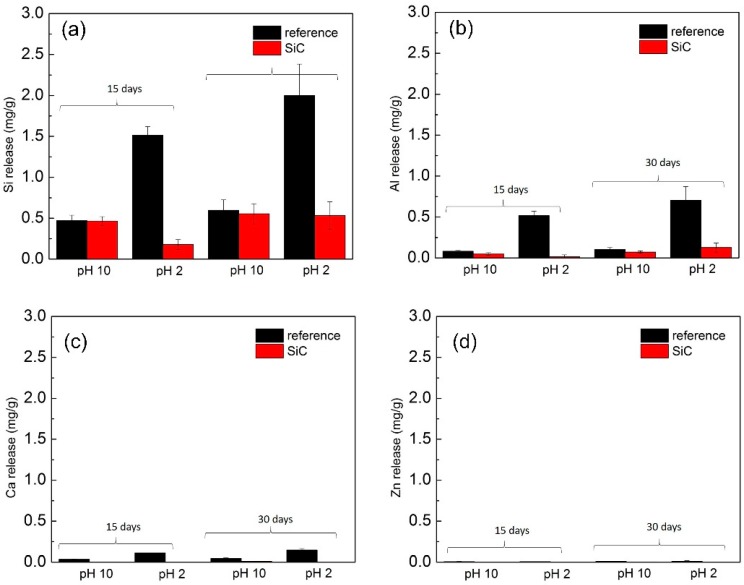
Ion release of (**a**) Si^4+^, (**b**) Al^3+^, (**c**) Ca^2+^, and (**d**) Zn^2+^ for SiC-coated and non-coated disks at pH 10 and pH 2 for 15 and 30 days.

The level of released ions from non-coated and SiC-coated disks in the solutions was analyzed after corrosion. The levels of ions released from non-coated disks are shown in Figure 2. The highest released ion was Si^4+^ in both buffer solutions, as this ion was the network former in the glass-ceramic and the main component in the SiC coating (Figure 2a). The overall Si^4+^ released across solutions and timepoints was significantly lower for SiC-coated disks (*p* = 0.014). The level of Si^4+^ released at pH 2 was marginally lower (*p* = 0.01) in SiC-coated disks than the non-coated disks, but was not significant at pH 10 (*p* = 0.699).

For the other ions, which are network modifiers, the overall release level was $Al^{3+}$ > $Ca^{2+}$ > $Zn^{2+}$. These levels of ions released in the non-coated disks were compared with the SiC-coated disks in Figure 2b,d. The SiC coating demonstrated a significantly resistive effect against corrosion in both environments for all ions. The overall release of $Al^{3+}$ for both solution types and across all time points was significantly less for SiC-coated disks (*p* = 0.003) (Figure 2b). When grouped by solution, *Al^3+^* ion release was significantly less with the SiC coating in pH 10 (*p* = 0.016) as well as in pH 2 (*p* = 0.010). When grouped by time point, there was less $Al^{3+}$ released at 15 days (*p* = 0.004) for SiC-disks than 30 days (*p* = 0.083). $Ca^{2+}$ was released significantly less with SiC-coated disks across all solutions and time points (*p* < 0.001) (Figure 2c). SiC-coated disks had significantly reduced $Ca^{2+}$ release in pH 10, and there was no detectable release of this ion for pH 2. Grouped by timepoint, $Ca^{2+}$ was released significantly less in SiC disks at 15 (*p* = 0.007) and 30 (*p* = 0.008) days. $Zn^{2+}$ was released significantly less in SiC-disks across all pH environments and time points (*p* < 0.0001) (Figure 2d). As with $Ca^{2+}$, there was almost no release of $Zn^{2+}$ ions from SiC disks in pH 2 (*p* = 0.006) and significantly less release at pH 10 (*p* = 0.002).

### 3.3. XPS Analysis

**Figure 3:**
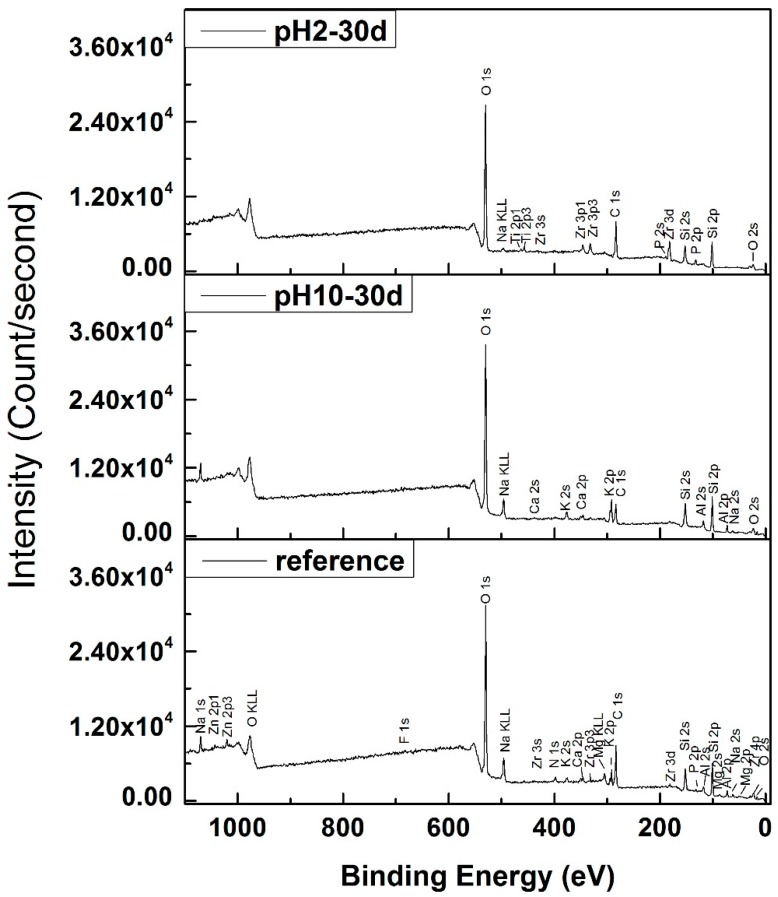
The X-ray photoelectron spectroscopy (XPS) survey for reference, corroded disks in pH 10 and pH 2 after 30 days.

The surface composition of the disks was analyzed using XPS (Figure 3 and Table 2). After immersion in pH 10, the spectrum had a slightly higher atomic ratio of Si, Al, Na, and K, whereas the Al, Na, K, and Ca were not detected on the corroded surface in pH 2 (Table 2). This is in agreement with the results of ions release (Figure 2). The ions were exchanged from the reacted surface with ions in the solution.

### 3.4. SEM Analysis

**Figure 4:**
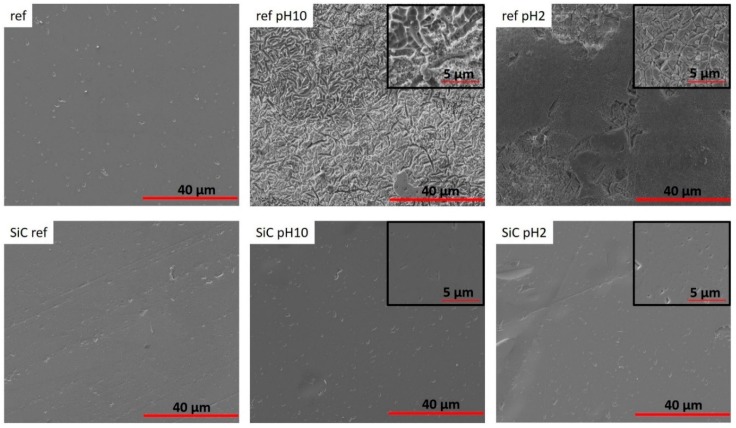
The images of reference (non-coating) and SiC-disks at 0 days (baseline) and 30 days after corrosion in pH 10 and pH 2. Scale bar: 40 μm and 5 μm.

The SiC coating was able to planarize the surface of glass-ceramic disks and seal surface porosities that were produced during fabrication. The images of SiC-disks before and after 30 days’ immersion are shown in Figure 4. The morphology of SiC-disks showed mostly good coverage and adhesion in pH 10 and pH 2. Surface roughness was evident in pH 10 and pH 2 non-coated disks after immersion, but was not too apparent on the SiC-disks immersed for the same time period. These findings are in agreement with weight loss and ICP data (Figure 1 and Figure 2). This demonstrated that SiC coating is an effective approach to improve the chemical stability of glass-ceramic materials by minimizing corrosion.

## 4. Discussion

The purpose of this study was to investigate whether this novel SiC coating will improve and exhibit significant corrosion resistance. Previous studies concluded that ceramic materials undergo a corrosion process when exposed to different pH environments. Corrosion produces surface degradation, which leads to roughening of the surfaces of ceramic crowns and wear of opposing enamel [1,10,50]. This in turn leads to plaque accumulation, secondary caries, and periodontal inflammation. Corrosion can also lead to discoloration of the restoration and a decrease in fracture strength of the glass-ceramic [13,14,51]. The results of this study demonstrated that SiC coating is an effective approach to improve the chemical stability of glass-ceramic materials by minimizing corrosion and maintaining a smoother surface (Figure 1, Figure 2 and Figure 4). The weight loss was significantly less for SiC-coated disks for all pH environments and time conditions. The application of the SiC coating on glass-ceramic can minimize these clinical sequelae from occurring.

Ceramic corrosion can occur through either ionic exchange of surface ions where network modifiers in the ceramic are leached and exchanged with protons or hydronium with those in solution, total dissolution of the glass network, or a combination of both. Previous studies demonstrated that pH 2 promotes ion exchange, pH 10 induces total dissolution with release of the network formers, and pH 7 is a combination of both processes [1,10]. During ion exchange, when alkaline ions are released into the solution, a silicon-rich surface can be formed on the surface, which can minimize the exchange process. The trend for weight loss in this study demonstrates a higher loss in pH 2 than pH 10, which is in contrast with results from another study [1]. One explanation could be that a new layer can also form in basic solutions, with $Ca^{2+}$ ions being dissolved on the surface [52]. This is evidenced by the presence of Ca peaks in the disks immersed in pH 10 solution, but not in pH 2 (Figure 3 and Table 2). The $SiO_{4}^{-}$ attaches to $Ca^{2+}$, and this limits the ion diffusion in and out of the glass-ceramic. Another possible explanation is that the composition of the material used in this study was different in that the glass-ceramic used in this study had more $Al_{2}O_{3}$ (Table 3) [53,54], which is typically used to improve chemical stability [22], as the alumina compound is known to have a small ionization constant in basic solution [55]. When $Al^{3+}$ ions are released into the solution during network dissolution, there is a possible formation of soluble ${\left[Al{\left(OH\right)}_{6}\right]}^{3-},$ $AlO_{2}{}^{-}$ in the solution [56], or $Al^{3+}$ precipitates could form on the surface to further inhibit the dissolution process. This was also confirmed by ICP analysis, where there was a smaller amount of $Ca^{2+}$ and $Al^{3+}$ released into solution from disks immersed in pH 10 compared with those immersed in pH 2. The XPS corroborates this by demonstrating Ca and Al peaks in pH 10 disks, but not in pH 2.

The SiC coating demonstrated a more protective effect on pH 2 by a greater reduction in weight loss between coated disks and non-coated disks compared with disks immersed in pH 10. This can be explained by the corrosion behavior of SiC. The reaction of SiC has been studied in aqueous solution through the electro-chemical method [56], where SiC produces a passivating layer of $SiO_{2}$ in acidic conditions. In contrast, the SiC dissolves into $SiO_{3}^{2-}$ in an alkaline solution. Therefore, the corrosion of SiC was much weaker in pH 2 than in pH 10, as confirmed by the results from this study. 

The SiC also demonstrated a resistive effect for the release of all ions, with the exception of Si^4+^, which had only marginally lower release. All ions demonstrated either marginally significant or significant effects of SiC at decreasing the release of ions into solution. A possible explanation for this is that the main component of the SiC films is Si^4+^, and as such, would be the first line of defense for the chemical attack from the buffer solutions. The weight loss data prove that not much else was leached from the disks coated with the SiC film. For the network modifiers, there was no detectable release of $Ca^{2+}$ or $Zn^{2+}$ at pH 2. $Al^{3+}$ in SiC-coated disks demonstrated a significant reduction in release overall, but this reduction was more apparent for pH 2 than pH 10. As mentioned previously, $Al^{3+}$ has a small ionization constant and an inhibition layer of $Al^{3+}$ precipitates could have formed on the surface.

One limitation of this study is that a constant immersion experiment was conducted to test the durability of this coating against different pH levels. This is not a true simulation of oral conditions because the oral environment has constantly changing pH resulting from different foods and the buffering capacity of saliva. However, the International Standards Organization standard for Dental Ceramic (ISO 6872) still employs constant immersion testing at pH 2.4 to test the chemical durability of ceramics [57].

A new testing methodology was previously introduced to determine the effect of changes in pH environment to simulate fluctuations intraorally resulting from dietary preferences [10]. This methodology demonstrated that chemical degradation is possibly being underestimated with current in vitro testing using constant immersion. This study determined how well SiC coatings can withstand extreme pH challenges in constant immersion. Testing SiC coatings in pH cycling conditions as well as the material’s fracture strength and abrasion resistance will be the next step in the continuum of developing novel coatings for predictable ceramic restorations.

## 5. Conclusions

In conclusion, a new and novel SiC coating can withstand the extreme pH conditions and improve the longevity of the restoration. This study demonstrated a significant protective effect on the chemical solubility of a glass ceramic veneer under different pH environments, as evidenced by decreased weight loss and ion release in solution. Ceramic composition played a role in the progression of the dissolution process. In addition, SiC coating provided the smoother surface after corrosion, which could minimize the plaque accumulation, secondary caries, and periodontal inflammation from occurring. This novel coating could be the next step in improving the longevity of ceramic restorations by increasing chemical resistance and minimizing fracture. Further studies of novel SiC coatings for dental applications are warranted to determine the corrosion resistance in pH cycling conditions, fracture resistance, and wear compatibility with enamel as in clinical relevance.

## Figures and Tables

**Table 1:** The compositions of the fluorapatite disks used in this study [34].

Composition	SiO_2_	Al_2_O_3_	Na_2_O	K_2_O	CaO	ZnO	ZrO_2_	P_2_O_5_	F	Other Oxides	Pigments
Wt %	57.0–62.0	12.0–16.0	7.0–10.0	6.0–8.0	2.0–4.0		1.5–2.5	1.0–2.0	0.5–1.0	0–6.0	0.2–0.9
Atomic %	58.6–51.6	14.5–15.7	13.9–16.1	7.8–8.5	2.2–3.5		0.7–1.0	0.4–0.7	1.6–2.6		

**Table 2:** The composition of reference, corroded pH 10 disks, and corroded pH 2 disks after 30 days.

Atomic Ratio	Si	Al	Na	K	Ca	Mg	Zn	Zr	N	P	F	Ti
ref	53.7	11.5	7.6	5.9	3.5	3.3	1.3	0.6	9.9	1.3	1.4	
OpH10	59.8	15.8	9.5	12.0	2.9							
NpH2	76.9		1.1					9.9		8.8		3.3

**Table 3:** Compositions of the disks from Ivoclar Vivadent scientific documentation for this study and another study [53,54].

Composition(wt.%)	SiO_2_	Al_2_O_3_	Na_2_O	K_2_O	CaO	ZnO	ZrO_2_	P_2_O_5_	F	Li_2_O	Other Oxides	Pigments
This study	57.0–62.0	12.0–16.0	7.0–10.0	6.0–8.0	2.0–4.0		1.5–2.5	1.0–2.0	0.5–1.0		0–6.0	0.2–0.9
Esquivel-Upshaw et al. 2013	60.0–72.0	2.0–8.0		10.0–23.0	1.0–10.5	8.5–20.0		0.5–6.0	0.1–1.0	1.0–5.0	5.0–10.0	0.0–0.3
